# Towards enhancing security of IoT-Enabled healthcare system

**DOI:** 10.1016/j.heliyon.2023.e22336

**Published:** 2023-11-15

**Authors:** Reyazur Rashid Irshad, Shahab Saquib Sohail, Shahid Hussain, Dag Øivind Madsen, Abu Sarwar Zamani, Abdallah Ahmed Alzupair Ahmed, Ahmed Abdu Alattab, Mohamed Mahdi Badr, Ibrahim M. Alwayle

**Affiliations:** a Department of Computer Science, College of Science and Arts, Sharurah-68341, Najran University, Kingdom of Saudi Arabia; b Department of Computer Science and Engineering, School of Engineering Sciences and Technology, Jamia Hamdard, New Delhi, India; c Innovation Value Institute (IVI), School of Business, National University of Ireland,Maynooth (NUIM), Maynooth, Co. kildare, W23, F2H6 Ireland; d USN School of Business, University of South-Eastern Norway, 3511 Hønefoss, Norway; e Department of Computer and Self Development, Preparatory Year Deanship, Prince Sattam bin Abdulaziz University, Al-Kharj 11942, Saudi Arabia

**Keywords:** Attribute based encryption, IoT-enabled healthcare system, Whale-based attribute encryption, Asymmetric key, Patient health record, ChatGPT

## Abstract

The Internet-of-Things (IoT)-based healthcare systems are comprised of a large number of networked medical devices, wearables, and sensors that collect and transmit data to improve patient care. However, the enormous number of networked devices renders these systems vulnerable to assaults. To address these challenges, researchers advocated reducing execution time, leveraging cryptographic protocols to improve security and avoid assaults, and utilizing energy-efficient algorithms to minimize energy consumption during computation. Nonetheless, these systems still struggle with long execution times, assaults, excessive energy usage, and inadequate security. We present a novel whale-based attribute encryption scheme (WbAES) that empowers the transmitter and receiver to encrypt and decrypt data using asymmetric master key encryption. The proposed WbAES employs attribute-based encryption (ABE) using whale optimization algorithm behaviour, which transforms plain data to ciphertexts and adjusts the whale fitness to generate a suitable master public and secret key, ensuring security against unauthorized access and manipulation. The proposed WbAES is evaluated using patient health record (PHR) datasets collected by IoT-based sensors, and various attack scenarios are established using Python libraries to validate the suggested framework. The simulation outcomes of the proposed system are compared to cutting-edge security algorithms and achieved finest performance in terms of reduced 11 s of execution time for 20 sensors, 0.121 mJ of energy consumption, 850 Kbps of throughput, 99.85 % of accuracy, and 0.19 ms of computational cost.

## 1 Introduction

**Fig. 1:**
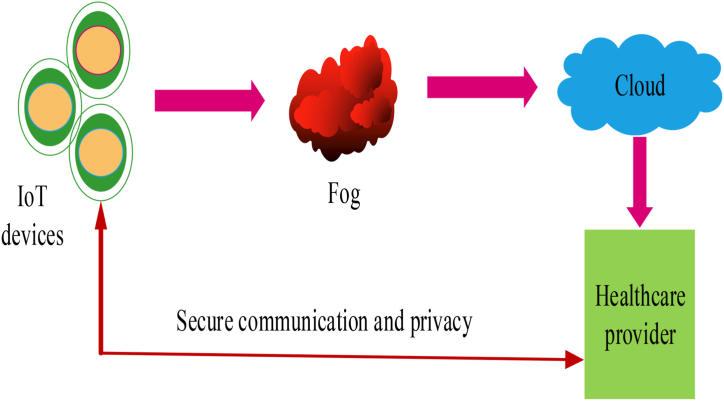
IoT for secure communication in healthcare system.

In recent years, the internet-of-things (IoT) healthcare systems emerge as the recent trend and allow for remote monitoring, data collecting and analysis, automation of cardiology, telemedicine, remote surgeries, and general healthcare management to improve patient healthcare [1]. The security of the patient health record (PHR) is particularly important due to the sensitive and valuable information contained in it related to the patient's history [2]. However, due to the widespread use of interconnected devices in digital healthcare systems, which allow for transmission to a centralized server to process the data, they are more vulnerable to potential breaches of healthcare information security [3]. In addition, healthcare has become a prominent area of applications in industrial sectors such as blood sugar levels, medical diagnoses, biomedical signals, heart rate, body parameters, and electroencephalography, all of which are monitored by multiple medical sensing devices and IoT sensors and devices [4,5]. In particular, the Internet of Things (IoT) is used to improve the quality of healthcare environments and overcome geographical barriers by automated production, remote monitoring, and providing up-to-date data to end users [6]. While the Internet of things (IoT) is employed in a variety of industries, its usage is particularly essential in the healthcare industry, where wearable devices, actuators, and sensors gather physiological data such as temperature, heart rate, electrocardiogram (ECG), blood pressure and so on [7,8]. The obtained data is then sent to nearby servers or devices, as illustrated in Fig. 1 shows an IoT-based healthcare system. Nevertheless, IoT healthcare is confronted with a critical issue of scalability and capacity, which results in large amounts of data being generated in real-time applications while also reducing the costs related to integration [9]. Accurate identification of IoT devices is directly related to the security and safety of healthcare data [10], so inadequate authentication provides opportunities for intruders or attackers to exploit the IoT devices and create false values [11].

In general, the Internet of Things promotes the development of applications in a multitude of new areas, such as medical aids, industrial automation, smart buildings, intelligent energy management, mobile healthcare, traffic management, and automation [12,13]. These applications generate large amounts of data is used to provide new services to companies, individuals, and government organizations for making informed decisions [14]. There are numerous advantages to deploying IoT applications in the healthcare context, including the identification and authentication of attacks, item tracking, and automatic data collection [15].

Moreover, sensors implanted in the human body to track blood pressure, heart rate, and temperature [16], and these monitoring results are received from the sensors and sent over the internet to a physician or healthcare provider for assessment and treatment [17]. However, for information centres with limited infrastructure, the increasing volume of electronic healthcare records, unstructured data, and diagnostic data presents never-before-seen difficulties. Continuous functioning, access, maintenance, and learning are required. More accessibility, worth, security, and evaluation are needed to make systems and information easier to handle. The operations of healthcare institutions must include data centres as essential components [18]. Moreover, building a privacy-preservation system to guard against unauthorized people accessing the personally identifiable information transactions is one of the biggest issues. A difficult problem involves providing a secure authorization transmission of data technique and keeping the integrity of the data while transferring the data across an IoT network [19]. The risk of issues with security, trustworthiness, dependability, secrecy, and other issues arises from the connectivity of devices [20].

Taking into account that IoT edge sensors are unattended components of the system, attackers have a greater possibility of hacking into the server and data by introducing unexpected delays, network failures, and injecting false data that can lead to incorrect decision-making [21]. As such, the IoT-enabled healthcare system is faced with major security and privacy risks, including user authentication and authorization; secure data transmission, and proper device management [22]. Furthermore, IoT devices require large amounts of energy with low processing capabilities and so a minor attack can have a major impact on the performance of the IoT edge sensors [23]. Consequently, real-time patient monitoring, decision-making systems, and financial data must be safeguarded against malicious attacks through the implementation of robust security measures such as encryption and secure access control [24]. The main contributions of this study can be summarized as follows.

● We have designed an IoT-based edge sensor-enabled patient monitoring architecture and presented a novel whale-based attribute encryption scheme that utilizes asymmetric key encryption to enable the transmitter and receiver to safely encrypt and decrypt patient health records acquired from IoT-based edge sensors.
● The suggested Whale-based Attribute Encryption process provides a secure means of encrypting attributes such as IP addresses, URLs, or MAC addresses of patient health record data and adjusting the whale fitness periodically to generate an appropriate master public and master secret key. This ensures security against unauthorized access and manipulation when transmitting the data to remote storage to be assessed for clinician decisions. Consequently, a novel WbAES is designed with the suitable parameters to secure the healthcare data from the third parties and secure data transmission.
● The proposed WbAES is validated using the patient health record (PHR) dataset acquired by IoT-based sensors, and multiple attack scenarios are generated and simulated using Python libraries. The performance of WbAES is then compared with the state-of-the-art Data Preservation and Lightweight Authenticated (DPLA) model [25], the (DL) based secure blockchain (DLSB) model [26], the SybilWatch Privacy-Aware Healthcare (SWPAH) technique [27], the Fog Computing based Three Tier Planning (FCTTP) model [28], the HEC based Cryptographic System (HECCS) [29], the Security based Instant Encrypted Transmission (SIET) and Privacy-Preserving Data Aggregation (PPDA) model [30], security algorithms in terms of execution time, energy consumption, throughput, accuracy, and computational cost.

The structure of the research paper is summarized as follows: Section 2 provides an overview of related works in healthcare security, Section 3 outlines the system model and associated problem statement, Section 4 explains the proposed methodology, Section 5 presents the results and discussion, and Section 6 provides a concluding summary of the designed model.

## 2 Related work

Numerous researchers in the literature have focused on optimization-based cryptography algorithms to enhance the security and privacy of the healthcare system; however, there are still some outstanding challenges that need to be addressed, such as high computational overhead, high-energy consumption, limited fault tolerance, and high traffic load [31].

Mohammed Amin et al. [25] proposed a data retention and lightweight authenticated scheme for supporting decentralized authentication amongst authorized devices. In their approach, they minimize the latency between paired devices and communication stats, using evaluation results of the developed model compared to other relevant approaches. The developed model shows excellent improvement when comparing parameters; however, it is highly energy-intensive.

Aitizaz Ali et al. [26] present a novel cryptographic system that uses a combination of deep learning and homomorphic encryption to enable and access data through search. Furthermore, the authors use an IoT dataset to evaluate the access control scheme, thereby increasing anonymity, security, and user behaviour tracking; however, the system's computational cost is comparatively considerable.

Vaishnavi and Sethukarasi [27] designed SybilWatch to detect a Sybil attack by leveraging the sensor properties of each node in their IoT-based smart healthcare system. Comparisons between data from real-world datasets and simulations illustrate that SybilWatch is more effective than existing methods when it comes to accuracy, efficiency, and scalability but requires a high storage capacity for storing an extensive collection of keys.

Shukla, Saurabh et al. [28] proposed a blockchain-based fog-computing model for authorization and authentication in the healthcare IoT, which utilizes a distributed authentication scheme backed by a consortium blockchain to provide privacy-preserving authentication and authorization. The main purpose of this research was to achieve secure data transmission, for which it utilized real-world healthcare datasets and simulations. Results show that the proposed method was successful in detecting malicious nodes with an accuracy of about 91 %, but the throughput rate was still relatively lower.

Kavitha et al. [29] proposed an improved authentication scheme based on hyper elliptic curve public key cryptography for IoT healthcare systems. This system utilizes a generalized group key agreement technique to enhance the security and authentication of the proposed system, and its efficacy was evaluated using rigorous security measurements and in comparison, with other related models. Results demonstrated that it outperforms existing methods, however, the fault-tolerant rate is relatively low due to decreased communication.

The majority of the research that were chosen for this study, according to Bubukayr MA et al. [32], focused on the main cybersecurity risks that affect smart phones and applications, such as malware attack, phishing attack, software failure, Dos attack, sniffer and spoofing attack, physical assault, etc. On the basis of the four components of a smartphone—the device, the application, the data, and the network connectivity—major cybersecurity vulnerabilities are also identified that undermine the system. Decide on a classification and ranking system for the system's biggest dangers.

Almaiah MA et al. [33] present an elliptic curve cryptosystem and hill cipher hybrid encryption approach (ECCHC) to change the Hill Cypher from a symmetric to an asymmetric strategy to improve security and efficiency and thwart hackers. The computation speed, security effectiveness, and simplicity are the key benefits of the suggested technique.

A comprehensive framework for mobile devices and applications-cyber security threat classifications was proposed Almaiah MA et al. [34] and incorporates the majority of cyber threat categories and guiding principles. This framework's major goal is to comprehensively detect cyber security dangers, demonstrate their possible effects, alert mobile users to these concerns, and empower them to take relevant precautions.

The Modified Particle Swarm Optimization (PSO) technique, which Al Hwaitat AK et al. [35] offer as a modified form of PSO, is intended to improve the identification of jamming assault sources across randomized wireless networks. The simulation findings demonstrate that the Modified PSO method in this study is quicker than other techniques at determining the position of the provided mobile wireless network at which the area of coverage is smallest and centralized.

With the use of voting, authorization, and heuristic identification techniques, Almaiah MA et al. [36] suggested a novel strategy for determining the best defenses to identify harmful and security risks while utilizing blockchain technology. In this architecture, the cluster head node uses Blockchain and the three detection mechanisms to find rogue sensor nodes. The final statistics revealed that 94.9 % of fraudulent messages were effectively discovered and detected during the test phase of our method.

Nevertheless, healthcare systems still need an efficient and secure approach to enhance the accuracy, throughput, energy consumption, execution time, and computational complexity while monitoring and controlling patient health records in real-time. This paper proposes a novel whale-based attribute encryption scheme (WbAES), which facilitates the encryption and decryption of data using asymmetric key encryption. The attribute encryption scheme transforms plain text into ciphertexts and modifies the whale fitness to generate a suitable secured keys, providing heightened security against unapproved access and manipulation.

## 3 The system model and the problem statement

**Fig. 2:**
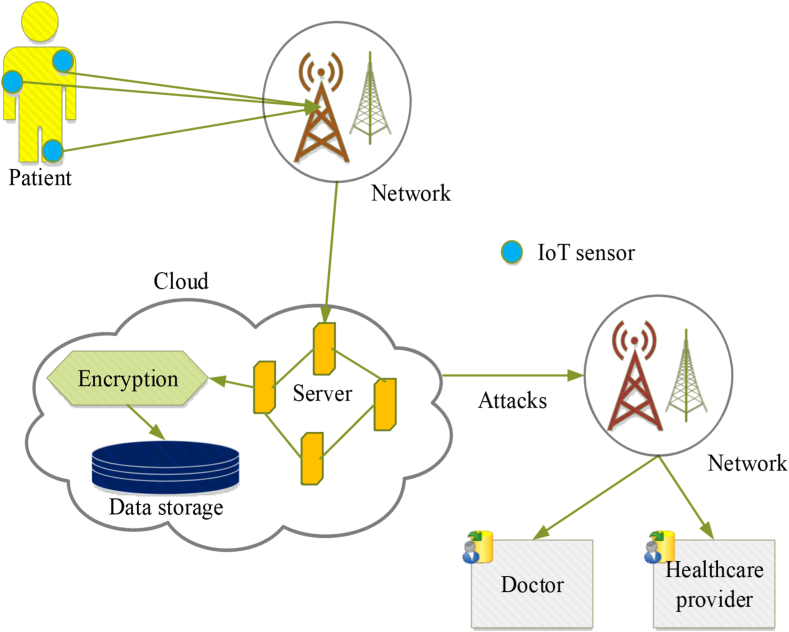
Outline of security framework in IoT-based healthcare system.

In recent years, IoT has been utilized for various applications in healthcare, allowing sensors and medical devices to exchange data autonomously and securely. IoT in healthcare has many benefits including improved patient outcomes, better quality care, and cost savings. This allows healthcare providers and doctors to review the transmitted data and take action as needed. While there have been several security-enhancing techniques developed for this purpose, they are still prone to high attack rates and lack of necessary security and privacy. These include a lack of secure communication protocols, vulnerability to cyber-attacks, and privacy concerns about sensitive health information being shared without the patient's consent. To ensure that secure communication protocols are established between different devices involved in the process, it is important to use encryption techniques that can provide strong authentication mechanisms as well as secure communication channels, as illustrated in the system model of the proposed WbAES in Fig. 2. It shows that wearable IoT edge sensors are attached to the human body to measure temperature, blood pressure, heart rate, and other metrics. The captured data is securely transferred to cloud servers over a wireless network and encrypted using cryptographic techniques to ensure its security.

Furthermore, the sensitive data is encrypted and stored in cloud-based data storage, allowing healthcare providers or doctors to access and use it in case of emergencies. The transmission of patient health records from IoT-edge sensors to the cloud and clinicians and doctors for decision-making purposes is especially delicate due to the vast number of networked intermediary devices and shared wireless communication channels. Additionally, the large volume of data generated and its processing, combined with the limited processing and energy capacity of IoT edge sensors, results in reduced data transmission accuracy and performance. To efficiently manage network traffic and protect the data through secured key generation, we propose optimization-based cryptography algorithms.

## 4 Proposed WbAES framework for IoT-based healthcare system

**Fig. 3:**
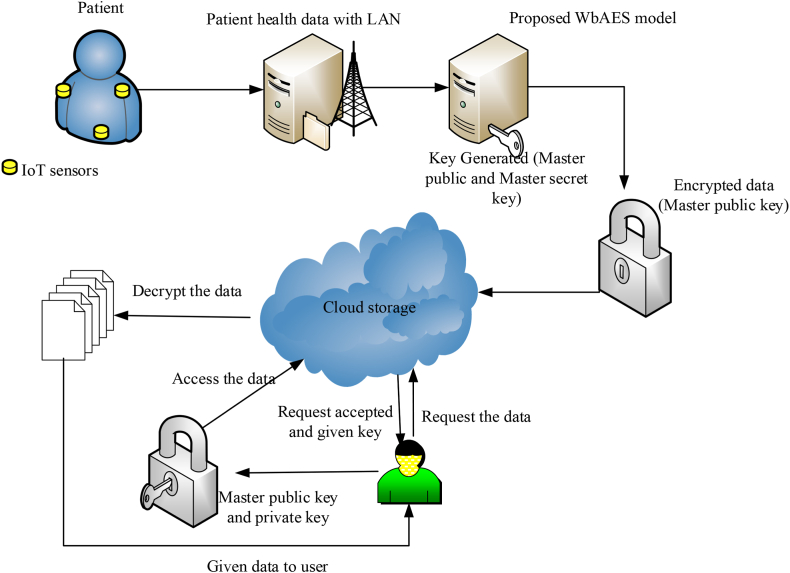
The conceptualization and the mechanism of securing the patient health record through the Proposed WbAES.

The methodology of the proposed system is illustrated in Fig. 3. The IoT sensors are placed in remote areas of patients and the healthcare data of patients are collected in the cloud via the LAN system. In this study, the data transmission has been secured by the proposed WbAES method. The proposed WbAES model utilizes a two-level encryption process to protect the transmitted healthcare data. The two-level process contains authentication and attribute-based encryption. The authentication level provides authorization in the network and ensures that only an authenticated person to access the data. This step validates the identity of authorized users before permitting access to the encrypted data. The attribute-based encryption step was employed to secure the healthcare data from unauthenticated access. The key generation phase involves the production of master keys for encryption and decryption processes. In the presented framework, the whale optimization algorithm (WOA) was utilized to generate the master public and secret keys. The WOA approach optimizes the selection and generation of the keys, providing security and improving the efficiency of the encryption process. The public and secret keys derived from the generated master keys are deployed for encryption and decryption processes, respectively. The master public key and secret key are used for the encryption process to convert the healthcare data into ciphertext. The private key, on the other hand, is used for the decryption process to convert the ciphertext back into plaintext for authorized access. This scheme also provides an extra layer of security due to its requirement of knowledge regarding specific characteristics related to a particular whale character. The encryption system also includes an access control mechanism, which is based on attributes and allows access only to those who have the correct attribute values. Finally, various attacks such as brute force assaults, phishing attacks, man-in-the-middle attacks, dictionary attacks, and social engineering attacks are launched in the system to evaluate the effectiveness and dependability of the WbAES model; the minimum deviation between the system performances before and after attacks demonstrates the effectiveness of the proposed model. The detailed functioning mechanism for each the components of the proposed WbAES model's is presented in the following sections.

### 4.1 The importance of the PHR data

PHR is critically important data that contains vital information about an individual's medical history, treatments, and medications. It plays a key role in providing healthcare professionals with the necessary facts to ensure accurate diagnosis and treatment of patients for maintaining their quality care. The main purpose of storing PHR is for providing continuous care via the IoT edge based sensors in monitoring the patient body temperature, blood pressure, heart rate, and so on. While cloud-based storage of patient health records can provide numerous benefits - such as easy access from any location, improved collaboration between healthcare providers, and greater efficiency in sharing data - there are also potential risks associated with storing sensitive information on the cloud. These include unauthorized access to confidential data by hackers or malicious users, accidental deletion or loss due to technical glitches, and lack of control over who has access to the data.

### 4.2 LAN in IoT-based healthcare system for PHR monitoring

Local area networks (LANs) play a key role in IoT edge-based sensor networks for monitoring patients in real-time and maintaining patient health records. They provide the necessary connectivity between devices, allowing data to be collected from sensors and transmitted to other parts of the network. This allows healthcare providers to monitor vital signs, track changes in medication dosages, or detect any potential complications that could arise due to an underlying condition. Furthermore, LANs allow healthcare providers access to the patient's medical history which can be used as a reference when making decisions regarding their care. By leveraging this technology, doctors can quickly identify trends or patterns that may indicate potential problems with a patient's health before they become more serious issues. The potential challenges and threats to an IoT edge-based sensor local area network include the risk of data leakage and unauthorized access, the inability to detect malicious activities, insufficient encryption protocols, and device malfunction categorized in the following four points.

⮚ **Unauthorized Access:** Unauthorized access to the network or data can compromise sensitive information and disrupt operations.
⮚ **Data Loss/Theft:** Loss of critical patient data due to theft, malicious attacks, or other means could lead to serious consequences for both patients and healthcare providers.
⮚ **Malware Attacks:** Malicious software such as viruses, Trojans, worms, etc., which can be used to gain unauthorized access or corrupt system files and applications can damage the entire network infrastructure and result in the loss of confidential data from connected devices.
⮚ **Denial-of-Service (DoS) Attacks:** Such attacks are designed to overwhelm a system with traffic so that legitimate users cannot access the services they need.

### 4.3 Key generation and data encryption using the WbAES framework

**Fig. 4:**
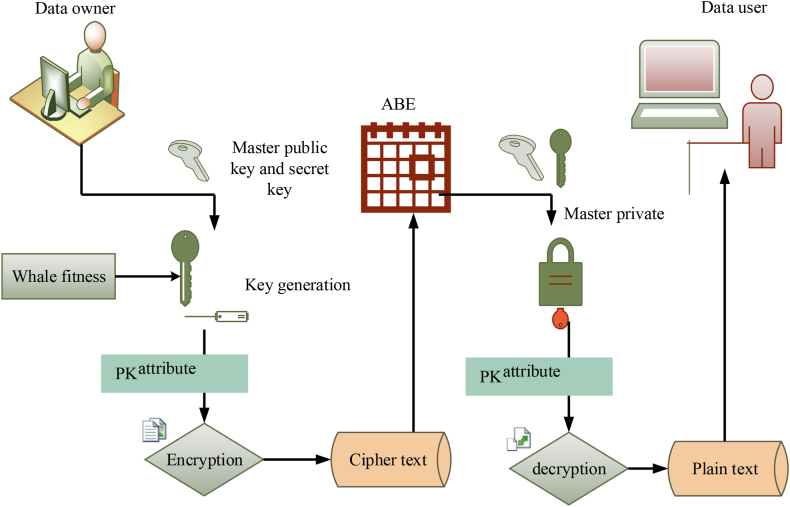
Encryption and decryption in the WbAES framework.

The primary goal of the proposed model is to secure data transmission and data storage in the medical field, where IoT edge sensors measure the vital signs of patients and transmit updated data to the cloud through connected local and wide area networks. In light of the security issues present in the IoT edge sensor-based network, it is essential to intelligently identify trustworthy authorities, data users, data owners, their privileges, and unauthorized users and their access. The main steps of the proposed security algorithm is key generation, encryption and decryption. The complete process for executing attribute-based encryption for medical data modifications is shown in Fig. 4. First, a user is authenticated and authorized to modify the medical data. Following this, an encryption key is produced using attributes related to the user and stored in a database. The encrypted data is then modified according to the user's request before being sent back to the database, where it is decrypted using the same encryption key and stored securely. Last, a new hash value is created, which can be used to validate that all alterations were done correctly and accurately, thus making sure of the accuracy of the modified medical data. The proposed WbAES model utilizes a robust two-level security process to secure healthcare data during transmission and storage. The first one is authentication level, which ensures authorized data access. The second one is encryption level in which the healthcare data was encrypted using the attribute-based encryption algorithm. This level safeguards the healthcare data during transmission. In the proposed methodology, the Whale optimization algorithm was employed for optimizing the key generation process in the encryption level. The WOA optimizes the key generation and selection processes, enhancing both security and efficiency. The WOA is a meta-heuristic approach inspired by the hunting characteristics of the humpback whales. The humpback whales hunt small fishes and krill for food. In case of key generation, these fishes and krill indicate the key features. The WOA approach dynamically changes the whale fitness and intends to find optimal key attributes, improving the key generation process.

For optimal key generation, the WOA employs two key strategies to optimize key attributes. The first one is exploitation strategy, which intends to locate the best solution relative to the target key variables. This is achieved by updating the position of the key in a spiral manner based on the fitness function. If the updated key position is close to the target, the other solutions adjust their position with the optimal one. The second one is exploration strategy, which aims to find the master keys using the random positions. The WOA enables its agents to explore the parameter solution and conducts a global search to estimate the finest master keys. After master key estimation, the secret key for encryption was determined. Once, the secret key is found, the healthcare data was encrypted with the public key to ensure data confidentiality and integrity during transmission. Finally, on the receiver end, the authorized user retrieves the original data by performing the revise operation with the secret key.

⁃ ***Setup phase*:** The trusted authorities are responsible for generating the master public and private secured keys, which are then utilized by data owners, such as healthcare professionals. The public key is used to encrypt data before transmission, and the secured key (asymmetric key) was employed for decryption by the authorized recipients. During the setup phase, the trusted authorities securely generate and distribute the master public key to data owners. The data owners encrypt the data attributes such as IP addresses, URLs, MAC addresses, etc., before transmission using the public key. After encryption, the ciphertext was transmitted to intended recipients. On the receiver end, the authorized users perform decryption using the secured key to convert the ciphertext into original text. Define the set of characteristics f={1,2,...,n}. Now, uniformly select a number j∈f at random from $X_{p}$ for each characteristic. Finally, select z from $X_{p}$ evenly at random. The public parameters $P_{ky}$ as was employed for encryption process. The set phase is mathematically expressed in Eqn. (1)

(1) $$H_{1}=w^{n},\ldots \ldots .H_{\left|f\right|}=w^{n\left|f\right|},X={e\left(w,w\right)}^{x}$$

The master key is expressed using eqn. (2),

(2) $$M_{ky}=n_{1},\ldots \ldots n_{\{f|},x$$

⁃ ***Key Generation phase*:** The whale optimization is included in the attribute-based security system for optimal key generation in encryption and decryption phase. This phase implements one of the hunting mechanisms that hunt small fishes and krill depending on foraging behaviour by producing separate bubbles in addition to the circles and attacking the target. Additionally, this step utilizes a random walk to the target zone to search for the prey with the highest fitness, which is then used to generate the appropriate key for the patient health record dataset transmission. The master key $M_{ky}$ and a list of properties *S* that characterize the key are inputs to the key generation algorithm. $S_{ky}$, a secret key, is the result. In the first phase, the master key is denoted by the $M_{ky}$ key, which is utilized to generate the public and secret key, and the whale fitness is dynamically changed to improve the performance of the key generation process. Assuming that the current best key solution is near the target key, other solutions change their positions with the best agent. The movement and the updation of key position are represented in Eqn. (3), (4).

(3) $$\vec{E}=\left|\vec{D}\cdot {\vec{M}}_{b}\left(n\right)-\vec{M}\left(n\right)\right|$$

(4) $$\vec{M}\left(n+1\right)={\vec{M}}_{b}\left(n\right)-\vec{B}\cdot \vec{E}$$

Where n is the number of current iteration, the coefficient vector is denoted as $\vec{B}$ and $\vec{D}$ the component should be in the range of [0,1]; this indicates that the value of the coefficient in these vectors should fall between 0 and 1. The position of the best master key is denoted as ${\vec{M}}_{b}\left(n\right)$ and the position vector is represented as $\vec{M}$; the position vector are defined within a specified range of [-1, 1]. The expression for coefficient vector is provided in Eqns. (5), (6).

(5) $$\vec{B}=2\vec{b}\vec{r1}-\vec{b}$$

(6) $$\vec{D}=2\vec{r2}$$

Where, the random sets belong [0,1] is denoted as $\vec{r1}$ and $\vec{r2}$.

***Key exploitation phase****:* Two strategies are developed to statistically simulate humpback whales' bubble-net activity. By lowering the value of " $\vec{b}$," this behaviour is produced. Throughout of iterations, a is reduced from 2 to 0. The spiral position of key is updated using eqn. (7), and the updation of master key is represented in Eqn. (8),

(7) $$\vec{E}=\left|{\vec{M}}_{b}\left(n\right)-\vec{M}\left(n\right)\right|$$

(8) $$\vec{M}\left(n+1\right)=\vec{E}.e^{cm}.\cos\left(2\pi m\right)+{\vec{M}}_{b}\left(n\right)$$

Where, $\vec{E}=\left|{\vec{M}}_{b}\left(n\right)-\vec{M}\left(n\right)\right|\text{,}$ the random number in [-1, 1] is denoted as m, c is constant for estimating the logarithmic spiral function. The Whale function moves around the best master keys within its limit. The probabilities of whale for key optimization is updated using eqn. (9)

(9) $$\vec{M}\left(n+1\right)=\left\{ \begin{array}{c} \begin{array}{cc} {\vec{M}}_{b}\left(n\right)-\vec{B}\cdot \vec{E} & ifP_{r} \end{array}<0.5\\ \begin{array}{cc} \vec{E}.e^{cm}.\cos\left(2\pi m\right)+{\vec{M}}_{b}\left(n\right) & if{P\geq 0.5}_{r} \end{array} \end{array}\right.$$

Where, the random number in [0,1] is denoted as $P_{r}$. If the master keys are not in this limit, then it moves to the exploration phases of WOA.

***Key exploration phase****:* The similar strategy based on the $\vec{B}$ set's variation may be used to the hunt for master keys. In actuality, humpback whales search at random based on where they are in relation to one another. As a result, we utilise $\vec{B}$ to compel the search agent to wander distant from a reference whale by giving it random values > 1 or <1. In the exploration phase, we update the position of an exploration agent based to a randomly selected search agent rather than the best search agent discovered thus far, in contrast to the exploitation phase. The WOA algorithm can conduct a global search thanks to this mechanism and | $\vec{B}$ | > 1's emphasis on exploration. The master keys searches are conducted at random using each other's positions and it is provided in Eqns. (10), (11)

(10) $$\vec{E}=\left|\vec{D.}\;{\vec{M}}_{r}\left(n\right)-\vec{M}\left(n\right)\right|$$

(11) $$\vec{M}\left(n+1\right)={\vec{M}}_{r}\left(n\right)-\vec{B}\cdot \vec{E}$$

If the criteria met, the iteration stops in final optimal keys otherwise return to the next iteration value. Finally, Equation (10) outlines the key generation process via the application of the fitness function.

(12) $$K_{g}=W\left(f\right)+\frac{M_{ky}}{P_{ky}}B_{n}\left(k_{0},k_{1},....k_{n}\right)$$

Where the terms W(f) denotes the fitness function of the whale, and $P_{ky}$ represents the generated public parameter. Furthermore, optimal public key generation is based on the selection of an appropriate random numbers r0,r1,r2........r(n). Besides, the other terms $B_{n}$ represents the bilinear pairing and the generated keys are denoted by $A_{0,}A_{1,}A_{2,}.....A_{n}$ for the healthcare data using attribution mechanism. Where, $k_{n}\in S_{ky}$ for varying master keys $M_{ky}$, $S_{ky}$ is represented as the secret key. Following the pool $M_{ky}$ of generated Keys, two random values α and β are chosen to generate the secured keys for encrypting the healthcare data and the patient's identification with $P_{ky}$, as well as the private key that will be shared to decrypt the data at the receiving end. Finally, provide the secured key to healthcare data to the medical entities $P_{ky}$ and patient's also public key $P_{i}$ is generated to encrypt the data, and master public keys and private key are generated to decrypt the data.

⁃ **Encryption (**$P_{ky},t,\alpha )$**:** In this encryption function the input PHR data t, $P_{ky}$ public parameter and α set of attributes are considered. The cipher text $c_{p}\left(t\right)$ is the output. The patient health care data has been encrypted with the use of generated optimal master keys expressed using equation. (13)

(13) $$En=(s,\tilde{E}=t,Z^{\alpha },{\left(E_{j}=H_{j}^{s}\right)}_{j\in \alpha }$$

Here in Equation. 11 the P(t) represents the plain text, t is the data and α is the attributes, and the $c_{p}\left(t\right)$ denoted ciphertext. Moreover, patient data are encrypted through the private key $P_{ky}$ by random locations in the cloud with the attributes represented by S(t). The decryption procedure from the ciphertext involves both the public key $Pu_{ky}$ and the private key $P_{k}^{s}$, which together change the ciphertext to the plaintext, as shown in Equation (14).

(14) $$de\left(s\right)=P_{ky},Pu_{ky}=S_{sk}^{i},{\left(S\left(t\right)=P_{i}\right)}_{i\in P_{ky}Pu_{ky}}=c_{p}\left(t\right)\to P\left(t\right)$$

The developed WbAES architecture is a monotonic access structure that is used to access the structure and forbids negative attributes. In the occurrence of any negative attribute, the proposed WbAES utilizes the specified attributes to detect attacks and thus prevent intrusions into the network, as demonstrated in Equation (15).

(15) $$A\left(d\right)=\frac{p_{ky}*Pu_{ky}-N\left(A_{n}\right)}{W\left(f\right)-P_{i}}$$

**Fig. 5:**
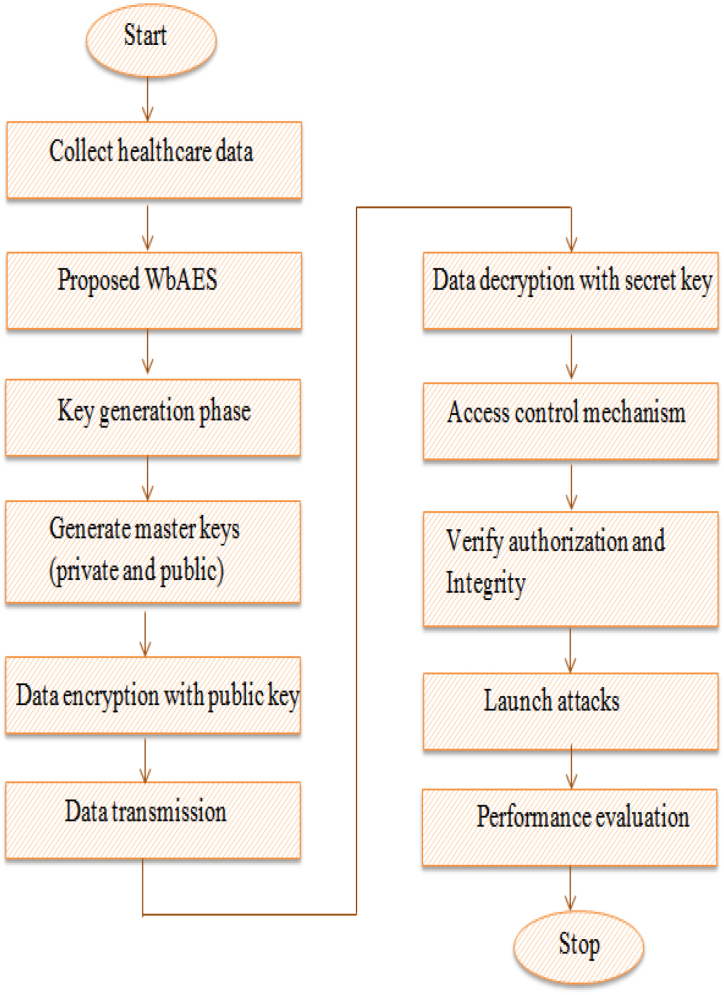
Flowchart of the proposed work.

Where $N\left(A_{n}\right)$ represents the negative attributes, which are identified by the WbAES and thus neglect the attacks leveraging these negative attributes. The detection of negative attributes is based on the matching of public and private keys, letting the server process them and accurately extract the unmatched negative attributes to identify attacks. The proposed model, therefore, enables a secure medium for the transmission of patient health record data via an IoT edge sensor-based cloud system through the categorization of attributes into positive and negative states. The pseudocode of the algorithm is presented in Algorithm 1 and its steps for identifying the negative attributed can be summarized as follows. Fig. 5 displays the workflow of the proposed work.

**Table:** 

Algorithm: WbAES
Start
{
*//Initialize healthcare dataset;*
*//Initialize WOA parameters, population size;*
User authentication ()
{
User attributes verification;//*user name, password*
}
WOA-key generation phase ()
{
*//Initialize key variables, iterations count i,* max *iteration;*
while i < max iteration
{
Exploitation phase ()
{
Calculate whale fitness;*//estimate the fitness of whale*
Update key position;
}
Exploration phase ()
{
Master key $M_{ky}$ generation;
Select master key;*/select the master key with greater fitness*
Generate secret key;*//create secret key from the master keys*
}
}
Encrypt data;*//perform data encryption using public key;*
Transmit data;*//transmit data to the storage*
Decrypt data;*//perform decryption using the secret key*
}
end

The negative attributes can be identified by the following methods.

1. Analysing the data packet size and type of data being transferred. If the packet size is too large or if it contains sensitive information, then it could indicate a potential attack.
2. Examining user patterns to see if there are any suspicious activities such as multiple failed login attempts or unauthorized access to resources.
3. Monitoring network traffic for unusual activity like large amounts of data being sent in a short period or connections to known malicious IP addresses and domains.
4. Scanning for vulnerabilities on devices connected to the system that could be exploited by an attacker, such as unpatched software, weak passwords, etc.
5. Categorize the attributes into positive and negative states to identify the attacks or any malicious activity.

## 5 Simulation results and discussion

In this module, the results of the presented security framework were analyzed by implementing it in the Python software. The performance of the developed model was examined by testing it with the real-time database collected from the 200 patients in IoT-based healthcare system. The outcomes were evaluated in terms of attack detection accuracy, energy consumption, throughput, execution time, and computational cost. Finally, the effectiveness of the designed model was estimated by comparing the results with the existing mechanisms. The performance enhancement score determined from the comparative assessment defines the robustness of the proposed method.

### 5.1 Simulation environment

The proposed WbAES framework is put into practice using the Python pyOpenSSL and pycrypto cryptographic libraries, then simulated against a total of 200 patient real-time data acquired by the IoT edge-based sensors, and assessed against the cutting-edge security algorithms in terms of accuracy, throughput, computational cost, energy consumption, and execution time. The proposed WbAES effectively transforms plain text into ciphertext by updating the whale's fitness and applying the ABE, which aids in improving the performance of key generation and securely transmitting to the cloud database. The following part discusses cutting-edge security algorithms and contrasts the results to emphasize the efficiency of the proposed WbAES.

### 5.2 Performance analysis

Numerous performance matrices, such as those measuring accuracy, throughput, energy consumption, computing cost, and execution time, are used to assess the proposed WbAES's efficiency. The evaluation is performed against numerous cutting-edge security algorithms, including the Data Preservation and Lightweight Authenticated (DPLA) model [25], the (DL) based secure blockchain (DLSB) model [26], the SybilWatch Privacy-Aware Healthcare (SWPAH) technique [27], the Fog Computing based Three Tier Planning (FCTTP) model [28], the HEC based Cryptographic System (HECCS) [29], the Security based Instant Encrypted Transmission (SIET) and Privacy-Preserving Data Aggregation (PPDA) model [30], respectively.

#### 5.2.1 Accuracy

The accuracy of a security algorithm is often judged based on how well the algorithm performs in accomplishing its intended function, such as preventing unauthorized access to data or systems, identifying hostile behaviour, and so on. The closeness of agreement between the outputs of the measured true values by the model is used to measure performance accuracy and demonstrates the effectiveness of the suggested technique, which ensures accurate information free of errors or mistakes. Thus, it is a degree of the computation, measurement, and specification outcomes to the standard or proper values. Table 1 compares the proposed WbAES's accuracy to that of the DPLA, SWPAH, and FCTTP algorithms while using varying numbers of IoT-edge sensors.

**Table 1:** Evaluating accuracy of different models different models with varying number of IoT sensors.

# of Sensors	Accuracy (%)			
	DPLA	SWPAH	FCTTP	Proposed
5	94.2	99.6	83	99.85
10	92.12	97.6	81.32	99.34
15	89.03	94	78	99.03
20	87.6	92.12	75.64	98.56

In the worst-case scenarios of networks with 5 and 20 IoT edge sensors, SWPAH and proposed WbAES achieved 99.6 % and 99.85 % respectively with a network of 5 sensors, while the proposed WbAES and SWPAH achieved 98.56 % and 92.12 % respectively with a network of 20 sensors. This showcases that the proposed WbAES is more accurate than DPLA and FCTTP, and is competitive with SWPAH even in worst-case scenarios, as represented in Fig. 5. Especially, the developed model achieved much better accuracy than other conventional models and attained an accuracy rate of 83.7 kB/s, which demonstrates the efficiency of the proposed WbAES model.

#### 5.2.2 Execution time

The total number of CPU cycles, also known as clock cycles, required for a processor to execute the instructions related to a specific program or process is referred to as execution time. This includes both the actual execution time taken to run instructions, as well as any wait times due to memory access, input/output activities, etc. Execution time is measured in hertz, which is the number of operations per second possible by the processor. A comparison of the execution time of the proposed WbAES against the DPLA, DLSB, FCTTP, and HECCS is presented in Table 2. According to Fig. 6, the proposed WbAES outperforms DPLA, DLSB, FCTTP, and HECCS in terms of execution time for all sizes of IoT edge-based sensor networks. Specifically, the DPLA technique used 40 ms of execution time when 5 sensors were employed; the DLSB technique achieved 20 ms; and the FCTTP model attained 27.5 ms. Furthermore, the HECCS model had 25 ms of execution time for 5 IoT edge-based sensors, while the DLSP model followed closely behind the proposed WbAES model when 20 sensors were used.

**Table 2:** Execution time validation over different number of IoT sensors.

# of Sensors	Execution time (ms)				
	DPLA	DLSB	FCTTP	HECCS	Proposed
5	40	20	27.5	25	5
10	47	24	29	29	7
15	53	28	33	34	9
20	56	32	37	39	11

**Fig. 6:**
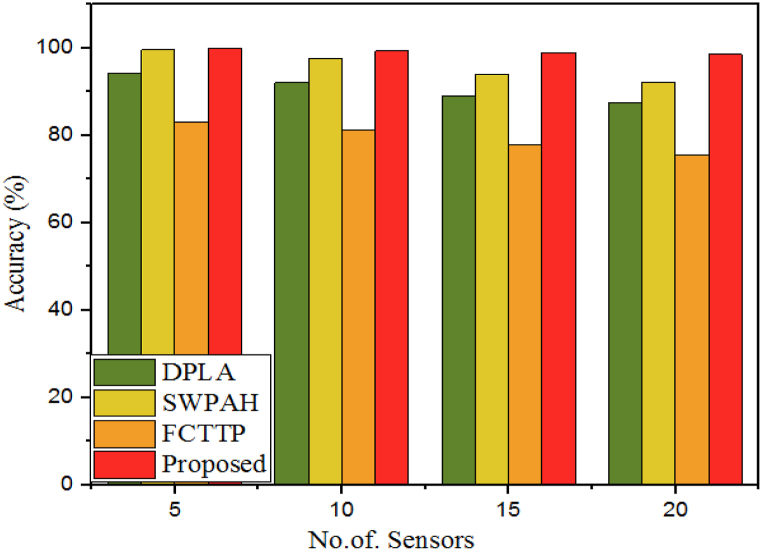
Comparison of accuracy of different models over varying number of IoT sensors.

#### 5.2.3 Throughput

The throughput of a security algorithm refers to the amount of data that can be processed by the algorithm within a certain period. This is typically measured in bits or packets per second and is used to gauge the performance of a particular computer system or network. It measures how long it takes an algorithm to complete an encryption or decryption task, with higher throughput being indicative of faster and more efficient security algorithms. The gained throughput performance of the designed model is compared with the SWPAH, FCTTP, PPDA, and proposed WbAES as detailed in Table 3. When the smallest network size with 5 IoT edge-based sensors was considered, the SWPAH model had a throughput rate of 260 Kbps, the FCTTP model achieved 318 Kbps throughput, and the PPDA model attained 432 Kbps throughput. The proposed WbAES had the highest throughput rate at 850 kbps. Similarly, for the largest network size of 20 sensors, the proposed WbAES had the highest throughput at 812 kbps versus the existing security methods, leading to improved system performance, as illustrated in Fig. 7.

**Table 3:** Throughput evaluation over different IoT sensors.

# of Sensors	Throughput (Kbps)			
	SWPAH	FCTTP	PPDA	Proposed
5	260	318	432	850
10	232	304	422	844
15	212	293	410	831
20	198	284	402	812

**Fig. 7:**
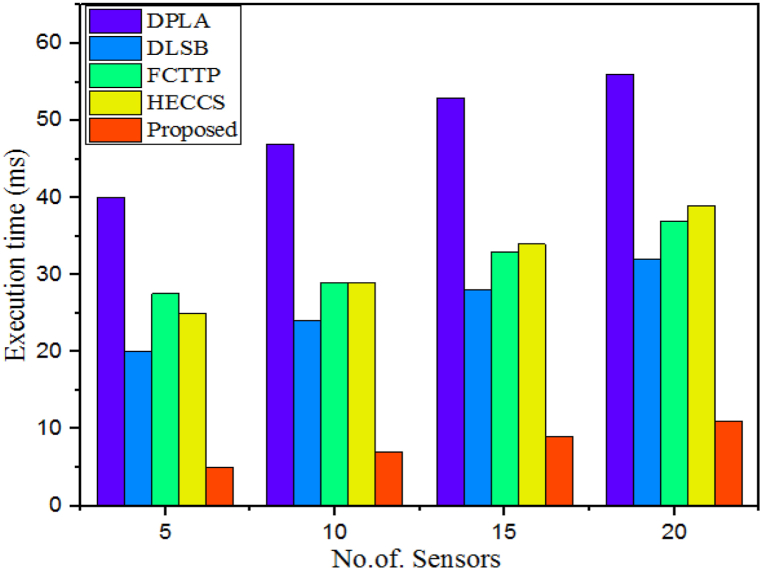
Comparison of execution time of various models over different number of IoT senors.

#### 5.2.4 Energy consumption

The energy consumption of an IoT-based sensor network is the total amount of energy consumed by all devices and sensors in the network. This includes both active and passive components, such as batteries, transmitters, receivers, processors, and other circuitry. The energy consumption of a sensor network can vary significantly depending on its size, type, application, and configuration. Measuring the energy consumption of a security algorithm helps to evaluate its performance in terms of efficiency, system longevity, and scalability. Energy consumption analysis is required to understand the algorithm's performance when transmitting PHR to cloud servers, which is necessary for healthcare data security. Measuring energy consumption by multiplying the wattage of the interconnected IoT edge-based sensor network of varying sizes can help evaluate the proposed algorithm's performance. Table 4 shows a quantitative comparison of the proposed WbAES's energy consumption in comparison to DLSB, FCTTP, and PPDA. The DLSB technique achieved an energy consumption of 0.000678wh, the FCTTP technique achieved an energy consumption of 0.0005051whthe PPDA achieved an energy consumption of0.000090wh, and the proposed WbAES achieved the least energy consumption of 0.000033wh when the network size of 5 IoT edge-based sensors was taken into consideration. For a network of 20 IoT edge sensors, the DLSB, FCTTP, PPDA, and suggested WbAES consumed 0.001015wh, 0.000888wh, 0.000263wh, and 0.000053wh, respectively. This suggests that when compared to the competing algorithms depicted in Fig. 8, the suggested WbAES exhibits superior performance with the least amount of energy consumption.

**Table 4:** Energy consumption evaluation with varying IoT sensor count.

# of Sensors	Energy consumption (Wh)			
	DLSB	FCTTP	PPDA	Proposed
5	0.000678	0.000505	0.000090	0.000033
10	0.000768	0.000592	0.000189	0.000038
15	0.000869	0.000768	0.000213	0.000046
20	0.001015	0.000888	0.000263	0.000053

**Fig. 8:**
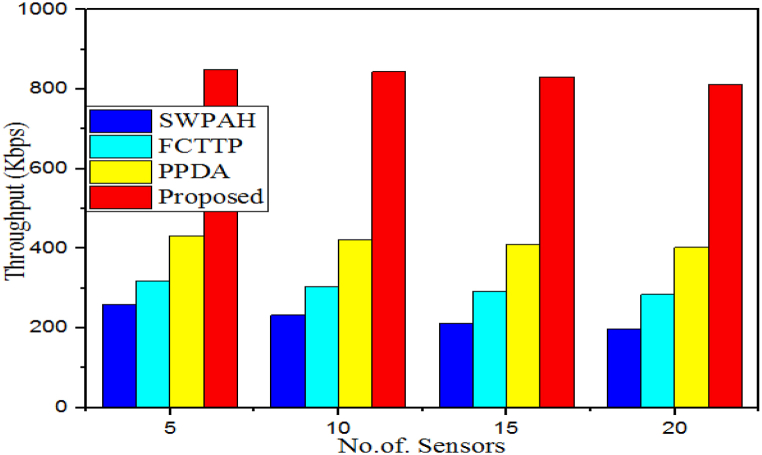
Validation of throughput of the presented model with different models over varying IoT sensors.

#### 5.2.5 Computational cost

The computational cost of security algorithms generally refers to the amount of computing power, time, and resources that are required to execute the algorithm. This includes things like processor cycles, memory, and storage space needed to run the security program or process. It also takes into account any additional overhead such as communication costs for verifying data integrity or encryption/decryption operations. The computational cost can vary greatly depending on the type of security algorithms being used and how they are implemented. A quantitative comparison of the computational cost of the proposed WbAES against the HECCS, SIET, and PPDA algorithm is detailed in Table 5.

**Table 5:** Computational cost validation over different sensor count.

# of Sensors	Computational cost (ms)			
	HECCS	SIET	PPDA	Proposed
5	1.28	0.25	0.31	0.12
10	1.98	0.36	0.47	0.14
15	2.54	0.54	0.62	0.17
20	3.12	0.77	0.73	0.19

**Fig. 9:**
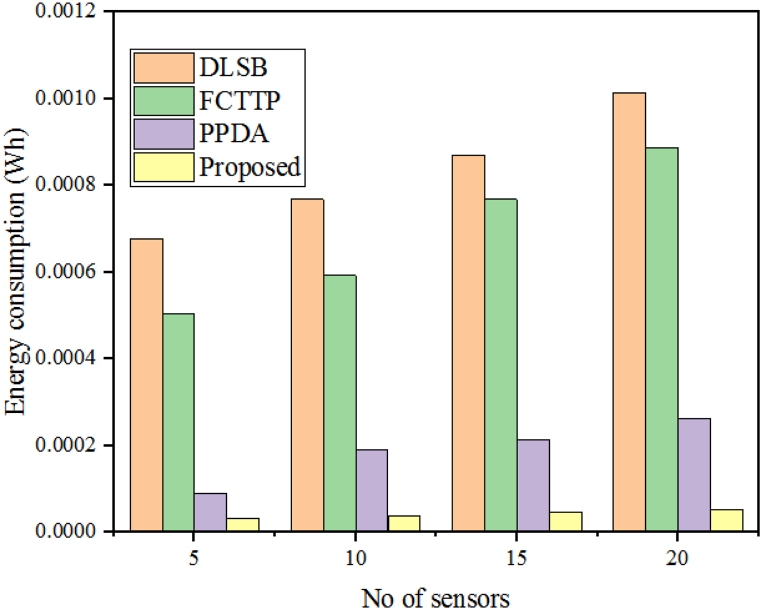
Comparison of energy consumption of different approaches with different sensor count.

**Fig. 10:**
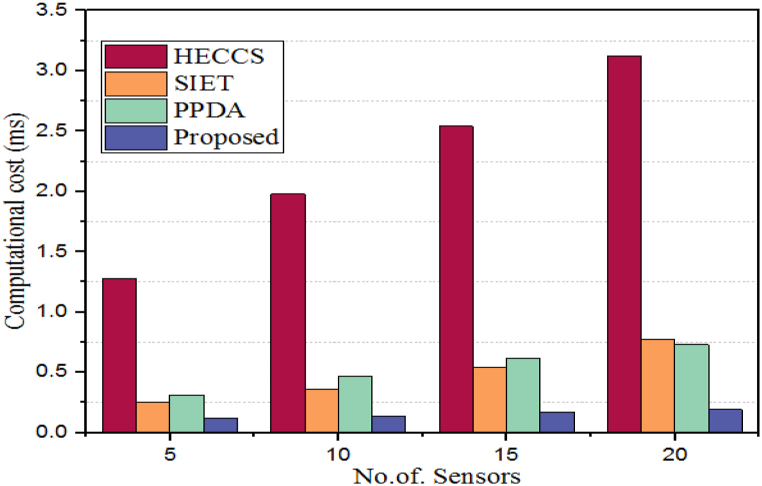
Comparison of computational cost of proposed with different models over different number of IoT sensors.

When compared to other security algorithms, the proposed WbAES algorithm has a lower computational cost. Fig. 9 demonstrates this as the computational costs for HECCS, SIET, PPDA, and WbAES at the smallest network size of 5 IoT edge-based sensors are 1.28 ms, 0.25 ms, 0.31 ms, and 0.12 ms, respectively. At the largest network size of 20 devices, the computational costs for the same algorithms are 3.12 ms, 0.77 ms, 0.73 ms, and 0.19 ms, respectively. The comparison of computational time is displayed in Fig. 10.

### 5.3 Discussion

In this article, a novel hybrid WbAES framework was presented to securely transmit the patients PHR data between two parties. The proposed work combines the benefits of whale optimization and the attribute encryption scheme. This integration of two approaches provides greater security and effectiveness in data transmission. The utilization of attribute-based encryption model in the presented work enables flexibility and offers fine-grained approach for data encryption. It selects the specific attributes for data encryption ensuring precise control over data access and improves the data confidentiality. Moreover, the utilization of Asymmetric master key enables a balance between the security and performance, ensuring efficient encryption and decryption operations for real-time applications. Furthermore, the integration of adaptive whale mechanism optimizes the encryption process by updating the whale fitness dynamically. In addition, the incorporation of additional security layer enables the system to detect the attributes, which imposes risk to data security. The IoT-based edge sensor-enabled patient monitoring architecture is secured by the proposed WbAES approach. The data of patients is transmitted to the receiver by encrypting the attributes of the system like, URLs, MAC addresses and IP addresses with the help of generated private key. The performance of the proposed WbAES is then compared with the state-of-the-art DPLA model [25], the DLSB model [26], the SWPAH technique [27], the FCTTP model [28], the HECCS [29], the SIET and PPDA model [30], security algorithms. The results of the simulation showed that WbAES outperformed existing security algorithms in terms of accuracy, energy usage, speed, expense, and run time. It was able to secure the PHR by using attribute encryption scheme conversion from plaintext to ciphertext and classifying attributes into positive and negative to detect any potential security lapses during the data transmission to the cloud. The execution time is shortened due to the reduced energy expenditure. From the comprehensive analysis of the performances, it is proved that the designed model detects the attacks or malicious threats accurately in short time, thus it minimizes the energy consumption, and computational time. Table 6 compares the performance criteria and security strategies in depth. As indicated in the table, the suggested WbAES has achieved an accuracy of 99.85 %, an energy consumption of 0.0000336wh and a throughput of 850 kbps. This shows that the proposed WbAES is highly reliable for delivering the PHR securely in the healthcare system over earlier models.

**Table 6:** Statistical comparative analysis of different models with the proposed model.

	Performance assessment		
Methods	Accuracy (%)	Energy Consumption (_wh_)	Throughput (Kbps)
SWPAH	99.6	–	260
FCTTP	83	0.000504	318
PPDA	–	0.000090	432
Proposed (WbAES)	99.85	0.0000336	850

## 6 Conclusion

In this paper, we introduced a new whale-based attribute encryption system (WbAES) for secure data transmission in healthcare framework. The presented method combines the advantages of WOA and ABE for protecting the data from security threats. The WOA integrated in the proposed method optimizes the key generation process, while the ABE offers effective encryption. The proposed WbAES was evaluated by utilizing patient health record (PHR) datasets collected from the Python libraries, which contains the information accumulated from IoT-based sensors, and various attacks. Further, the results of the study are assessed and compared with the existing security mechanisms like DLSB**,** FCTTP, and PPDA in terms of processing speed, energy consumption, rate of data transmission, precision, and computational cost. Consequently, the generated model's assessment results had remarkable performance, with an energy consumption rate of 0.121 mJ, a throughput of 850 kbps, and an attack detection accuracy of 99.85 %, compared to other existing methods, leading to an effective system for protecting patient health record data as it is transferred to the cloud system through an IoT edge-based sensors network. From the intensive analysis of the proposed model, it is evident that the integration of whale optimization and the attribute encryption scheme provided secure data transmission between the two parties, and mitigate the security threats in IoT-based healthcare system. This framework is more suitable for real-world healthcare applications such as patient data privacy preserving, secure healthcare data exchange, remote and telemedicine healthcare, medical researches, healthcare data analysis, electronic healthcare units, etc. In these application, the proposed framework enables secure communication and data protection from the third parties by providing integrity and confidentiality in the IoT-based healthcare platforms. However, the attribute-based encryption utilized in the presented model limits the attack prediction coverage; the presented approach does not cover other important attributes in the patient health records, which reduces the model efficiency in attack detection. Also, this proposed model cannot provide an accurate selection for certain relevant attributes, which reduces the reliability and security of the encryption system. Moreover, the attribute-based encryption model induces computational overhead, when applied to large number of attributes and complex databases. To resolve the above mentioned issues, the future research work must concentrate on designing intelligent algorithms to enable accurate and automated selection of the most relevant attributes for ciphertext conversion. Moreover, developing the multi-objective optimization-based encryption scheme improves the attribute selection and enhances the scalability and reliability performances. In addition, the optimization of the encryption algorithm increases the attribute coverage, thus the generalizability can be improved. Furthermore, incorporating the artificial intelligence in IoT-based system enables real-time monitoring and provides automatic detection and mitigation of attacks. To investigate this further, large language models, like ChatGPT, etc. can also be explored and maybe useful for the researchers working in this area.

## Data availability statement

Data will be made available on request.

## CRediT authorship contribution statement

**Reyazur Rashid Irshad:** Conceptualization, Data curation, Formal analysis, Investigation, Methodology, Software, Writing – original draft, Writing – review & editing. **Shahab Saquib Sohail:** Conceptualization, Formal analysis, Investigation, Methodology, Project administration, Supervision, Writing – original draft, Writing – review & editing. **Shahid Hussain:** Investigation, Methodology, Resources, Validation, Writing – original draft, Writing – review & editing. **Dag Øivind Madsen:** Project administration, Resources, Writing – review & editing. **Abu Sarwar Zamani:** Resources, Validation, Writing – original draft, Writing – review & editing. **Abdallah Ahmed Alzupair Ahmed:** Resources, Validation, Writing – original draft, Writing – review & editing. **Ahmed Abdu Alattab:** Resources, Validation, Writing – original draft, Writing – review & editing. **Mohamed Mahdi Badr:** Resources, Validation, Writing – review & editing. **Ibrahim M. Alwayle:** Resources, Validation, Writing – review & editing.

## Declaration of competing interest

The authors declare that they have no known competing financial interests or personal relationships that could have appeared to influence the work reported in this paper.
